# Supplementary material for: Stacking Model‐Based Classifiers for Dealing With Multiple Sets of Noisy Labels
Source: Biom J. 2025 Mar 12;67(2):e70042. doi: 10.1002/bimj.70042 (PMC11898607; doi:10.1002/bimj.70042)
Supplement: Supplementary file 1 — Supporting Information [file BIMJ-67-e70042-s001.zip › density-based-ensemble-model-main/competitors_real_data/netlab-master/netlab-master/help/conjgrad.htm]

Netlab Reference Manual conjgrad


# conjgrad

## Purpose

Conjugate gradients optimization.

## Description

`[x, options, flog, pointlog] = conjgrad(f, x, options, gradf)` uses a
conjugate gradients
algorithm to find the minimum of the function `f(x)` whose
gradient is given by `gradf(x)`. Here `x` is a row vector
and `f` returns a scalar value.
The point at which `f` has a local minimum
is returned as `x`. The function value at that point is returned
in `options(8)`. A log of the function values
after each cycle is (optionally) returned in `flog`, and a log
of the points visited is (optionally) returned in `pointlog`.

`conjgrad(f, x, options, gradf, p1, p2, ...)` allows
additional arguments to be passed to `f()` and `gradf()`.

The optional parameters have the following interpretations.

`options(1)` is set to 1 to display error values; also logs error
values in the return argument `errlog`, and the points visited
in the return argument `pointslog`. If `options(1)` is set to 0,
then only warning messages are displayed. If `options(1)` is -1,
then nothing is displayed.

`options(2)` is a measure of the absolute precision required for the value
of `x` at the solution. If the absolute difference between
the values of `x` between two successive steps is less than
`options(2)`, then this condition is satisfied.

`options(3)` is a measure of the precision required of the objective
function at the solution. If the absolute difference between the
objective function values between two successive steps is less than
`options(3)`, then this condition is satisfied.
Both this and the previous condition must be
satisfied for termination.

`options(9)` is set to 1 to check the user defined gradient function.

`options(10)` returns the total number of function evaluations (including
those in any line searches).

`options(11)` returns the total number of gradient evaluations.

`options(14)` is the maximum number of iterations; default 100.

`options(15)` is the precision in parameter space of the line search;
default `1e-4`.

## Examples

An example of
the use of the additional arguments is the minimization of an error
function for a neural network:

```
w = quasinew('neterr', w, options, 'netgrad', net, x, t);
```

## Algorithm

The conjugate gradients algorithm constructs search
directions `di` that are conjugate: i.e. `di*H*d(i-1) = 0`,
where `H` is the Hessian matrix. This means that minimising along
`di` does not undo the effect of minimising along the previous
direction. The Polak-Ribiere formula is used to calculate new search
directions. The Hessian is not calculated, so there is only an
`O(W)` storage requirement (where `W` is the number of
parameters). However, relatively accurate line searches must be used
(default is `1e-04`).

## See Also

`graddesc`, `linemin`, `minbrack`, `quasinew`, `scg`

---

**Pages:**
Index


---

Copyright (c) Ian T Nabney (1996-9)
